# DRUGS System Enhancing Adherence of Chinese Surgeons to Antibiotic Use Guidelines during Perioperative Period

**DOI:** 10.1371/journal.pone.0102226

**Published:** 2014-08-22

**Authors:** Zhifu Yang, Peixi Zhao, Jingwen Wang, Liping Tong, Jinyi Cao, Yun Tian, Zhanpeng Yao, Jingbo Wang, Yanrong Zhu, Yanyan Jia, Aidong Wen

**Affiliations:** 1 Department of Pharmacy, Xijing Hospital, the Fourth Military Medical University, Xi'an, Shaanxi, People's Republic of China; 2 Department of Pharmacy, Shaanxi Province Tumor Hospital, Xi'an, Shaanxi, People's Republic of China; 3 Department of Medical Affair, Xijing Hospital, the Fourth Military Medical University, Xi'an, Shaanxi, People's Republic of China; California Department of Public Health, United States of America

## Abstract

**Objective:**

Irrational use of antimicrobial agents for preventing postoperative SSIs is a common phenomenon in China, which results in more bacterial resistance, higher hospital infection rates, extra costs of antimicrobial agents. The aim of the study is to evaluate the effect of Drug Rational Usage Guidelines System *(DRUGS)* on the surgeon's prescription behavior of antimicrobial agents.

**Methods:**

10 common surgical operations which included 1543 cases (where 778 cases using paper-based guidelines and 765 cases using *DRUGS*) were selected and their demographic and clinical data were collected. The selected operations include thyroid resection, breast mass resection, myomectomy, etc. The evaluation criteria were antibiotic administrative categories, the time of initial dose, duration of administration, length of stay, the costs of antibiotics, SSIs and drug adverse reactions(ADR).

**Results:**

The antimicrobial agents were mostly administrated within 0.5 h to 2 h before incision, 656 patients (85.75%) were intervened with *DRUGS* and 256 (32.90%) with paper-based guidelines according to the protocol. For the clean wounds incision, 547 patients (91.62%) were within 24 h of withdrawal antibiotics with using paper-based guidelines versus 91 (14.79%) with using *DRUGS*. A total of 19 kinds of antibiotics were used in the 1543 cases. The leading three on the list of frequency were piperacillin and sulbactam sodium, cefathiamidine and cefoperazone. While after the intervention, the list of frequency changed to cefazolin, cefathiamidine, cefoperazone. The average hospital stay was (7.00±4.31)d with paper-based guidelines and (2.54±1.57)d with *DRUGS*, respectively. The average cost of antibiotics was ¥(3481.36±2584.46) with paper-based guidelines and ¥(1693.39±1478.27) with *DRUGS*, respectively. However, there were no significant differences in the incidence of SSIs and ADR between two groups.

**Conclusion:**

In this study, the increased availability of antibiotic guidelines at the time of drug ordering, combined with *DRUGS*, was associated with an enhanced surgeon adherence to guidelines.

## Introduction

Surgical site infections (SSIs) account for approximately 15% of nosocomial infections and are associated with prolonged hospital stays and increased cost [1]. According to the evidence-based guidelines for the prevention of SSIs published by the Centers for Disease Control (CDC), strategies for the prevention of SSIs are based both on reducing the risk of bacterial contamination and improving the patient's defense against infection. One of the most important interventions in preventing surgical site infections is the optimization of antimicrobial prophylaxis. Surgical antibiotic prophylaxis (SAP) is designed for achieving effective antibiotic concentration at the time of initial surgical incision, and maintained throughout the vulnerable period of the procedure, which is between skin incision and skin closure [2]. Many results demonstrated that prophylactic antibiotics administered preoperatively significantly reduce the incidence of SSI for patients [3].

In recent decades, guidelines have been developed to promote a rational use of SAP [4], [5], [6]. However, in many countries the use of SAP adhering to the guidelines is not always optimal [7], [8], [9]. In developing countries, less than 40% of patients in the public sector and 30% in the private sector are treated following clinical guidelines [10]. In China, the therapeutic treatment consisted with guidelines is severely lacking, which maybe the major reason for the high cost of medical service [11], [12]. So the key point to prevent SSIs is to improve the dependence on the guidelines for the prevention of SSIs. In addition, some studies have reported that making guidelines or decision supports can supply therapeutic information to prescribers ‘just-in-time’ to influence the decision at the time of clinical decision, so that surgeon is likely to apply it [13], [14], [15].

In Xijing hospital, Drugs Rational Usage Guideline System (*DRUGS*) was designed by the Fourth Military Medical University and Beijing TYT software technology Co, Ltd (http://www.tytsoft.com.cn/). Specialists on the field of medication and healthcare management would be organized to establish databases involving all aspects of rational drug use guidelines according to the national or international newest guideline in antimicrobial prophylaxis use in surgery [5], [16], [17]such as Chronic obstructive pulmonary disease [18], [19], [20] and their clinical experiment, The Chinese Medical Association of corresponding field were organized to evaluate these rational drug use guidelines. And then, the guidelines were inserted into the Computerized Physician Order Entry System. Further, the guidelines received consensus from all the head of the surgical department before being implemented in the hospital. It will provide therapeutic information to prescribers ‘just-in-time’ to influence the decision at the time of clinical decision, and update medical and drug knowledge to achieve effective supervision and management. The aim of this study is to investigate the effect of the DRUGS on reducing medication errors of antibiotics in 10 kinds of SAP.

## Methods

### Study practices

Xijing hospital is a university-affiliated hospital comprising more than 1500 beds in surgery including orthopedic surgery, general surgery, liver and gall surgery, stomach intestine surgery, obstetrics and gynecology. In this hospital, the most frequent antibiotic prophylaxis of surgical operations include thyroid resection, breast mass resection, inguinal hernioplasty, total hip replacement, uterine-incision delivery, myomectomy, excision of meniscus of knee, discectomy, internal fixation of fracture, and laparoscopic cholecystectomy.

Most of Class I/Clean wounds incision is an uninfected surgical wound in which no inflammation is encountered, which infection pathogen is *Staphylococcus aureus (S. aureus and coagulase-negative staphylococci)*. First-generation cephalosporins are generally recommended for prophylaxis; Class II/Clean-contaminated wounds, a surgical wound in which the respiratory, alimentary, genital, or urinary tracts are entered under controlled conditions and without unusual contamination. Specifically, surgical procedures involving the biliary tract, appendix, vagina, and oropharynx are included in this category, provided no evidence of infection is encountered and no major break in technique occurs. Since these technique such as percutaneous endoscopic gastrostomy (PEG), laparoscopic cholecystectomy(LC) and endoscopic retrograde cholangiopancreatography(ERCP) were already employed in the surgery of the abdominal cavity organs, and the wounds incision were mainly infected with the gram-negative Enterobacteriaceae the first or second generation cephalosporins is recommended to inhibit the infection. S aureus was responsible for more SSIs than any other microorganism. The first generation of cephalosporins are effective against *S. aureus*,have narrower spectrum of antibacterial activity, are safe and not so expensive as well. Therefore, these agents are preferred to most surgical procedures. According to the requisition of special actions of antimicrobial agents launched by the Ministry of Health of China in 2013 cefazolin was recommended as antimicrobial prophylaxis for Class I/Clean wounds incision and cefuroxime for Class II/Clean-contaminated wounds, and lincomycin or aztreonam was the alternatives for patients allergic to cephalosporin.

For the purpose of prophylaxis, and inoculums is minimal/none, antibiotics are administrated only for the duration of surgical procedure. If no infection exists, prophylaxis is preferred to surgery single dose. If the procedures last more than 3 hours, readministration is recommended when the antibiotic has a relatively short half-life. For example in prolonged procedures, an additional intraoperative dose of cefazolin should be given at 3 hours based on serum half-life (t_1/2_) data. Optimal time to give an antibiotic is 0.5–2 hour before incision. and optimal duration remains as an area of discussion. For many surgical procedures, a single dose is adequate to prosthetic device. As part of the development of the computerized order entry system of the hospital, *DRUGS* was integrated into the antibiotic order entry. At the beginning of surgical antimicrobial prophylaxis, in order to improve surgeon adherence to antibiotic guidelines, an intervention was decided by the Pharmacy and Therapeutics Committee(PTC)and received consensus from all the head of the surgical department before being implemented in our hospital. The study was approved by the institutional Ethics Committee for the use of humans in research of Xijing hospital(NO. LL-2012-026) and obtained their written informed consent where this the consent procedure were approved by ethics committees. The intervention was embedding the guidelines in the computerized physician order entry system of the hospital so that they were available online to the prescriber at the time of antibiotic ordering. Additionally, reinforcement was performed by the pharmacy department every 3-month period through an educational round with newly arrived physicians in the hospital. Although there was,no clinical pharmacist in the wards.

### Data collection and evaluation

The information and data of inpatients were collected through *DRUGS*, which was a computer-assisted antibiotic order system in the hospital, and covers all 10 antibiotic prophylaxis of surgical operations between September and December 2012. Collected information for each patient being on surgical operations included the patient's demographic data, the characteristics (mode of acquisition, clinical risk factors) of the possible infection, and data of the related antibiotic order (name of the antibiotic, daily dosage, planned duration of prophylaxis).

All the cases of antibiotic prophylaxis of the 10 surgical operations mentioned above were supervised and their data were recorded during the hospital stay. There were totally 1543 cases, including 778 episodes of surgical operations followed the paper-based guidelines, and 765 adhered to *DRUGS* (computerized guidelines).

Conformity of antibiotic orders to guidelines, reflecting surgeon adherence, was evaluated independently by the members of the antibiotic clinical therapeutics and supervision group according to the guideline for prophylaxis use of antibiotics:

the choice of antibiotic categories;
the time of initial dose of antibiotic;
the duration of administration.

For pharmacoeconomics, 10 cases were evaluated independently by two members of the PTC according to the following criteria:

the length of stay;
the costs of antimicrobial agents;
surgical site infections and drug adverse reactions.

Those antibiotic regimens with discordant evaluations between the reviewers were discussed to reach an agreement.

The chi-square (χ^2^) test was used for categorical variables including the time of the initial dose of antimicrobial agent, the disposition and kinds of SSIs. The student's *t* test was applied to analyze the continuous variables including the duration of administration in each surgical department, average cost and hospitalization days in DRUGS and paper-based guidelines. (For the variance nonhomogeneity, the *t*' test was used alternatively) Statistical analyses were performed by SPSS17.0 for Windows (SPSS Inc., Chicago, IL). Two-tailed *P*<0.05 was considered statistically different.

Approval for such an intervention and for assessing its effects through reviewing antibiotic drug regimens was obtained from the PTC. The present study was conceived as a doctor -nurse quality improvement project.

## Results

### Characteristics of patients

1543 surgical operations were recorded totally, where 778 followed the paper-based guidelines and 765 followed *DRUGS*. Mean age of the patients was 39.72±9.15 year's old versus 38.55±11.24 year's old for paper-based guidelines and *DRUGS*, respectively. Sex ratio (Male/Female) was 1.10 and 1.24, respectively. More than one episode of surgical operations occurred in 60 patients (Table 1).

**Table 1:** Characteristics of patients in each period before and after intervention.

State of patient	Paper-based guidelines	DRUGS
Sex (Male/female)	407/371	424/341
Year (years), (mean±SD)	39.72±9.15	38.55±8.58
Weight(kilograms), (mean±SD)	58.47±3.25	57.56±5.71

### The disposition and kinds of *SSIs*

All information was obtained from five clinical departments, including Department of orthopedics (717cases), general surgery (265 cases), liver and gall (139 cases), stomach intestine surgery (159 cases), gynaecology and obstetrics (263 cases). The disposition and kinds of *SSIs* are shown in Table 2. No statistic difference was found on the disposition and kinds of *SSIs* between the two groups by *χ* ^2^ test (*P* = 0.923)

**Table 2:** The disposition and kinds of *SSIs* in each period before and after intervention.

Department/Kinds of *SSIs*	Case (%)	
	Paper-based guidelines	*DRUGS*
General surgery		
Thyroid resection	53 (6.81)	47 (6.14)
Breast mass resection	85 (10.93)	80 (10.46)
Stomach intestine surgery		
Inguinal hernioplasty	83 (10.67)	76 (9.93)
Orthopedics		
Total hip replacement	90 (11.57)	102 (13.33)
Excision of meniscus of knee	89 (11.44)	76 (9.93)
Discectomy	95 (10.67)	87 (11.37)
Internal fixation of fracture	83 (10.67)	95 (12.42)
Gynaecology and obstetrics		
Myomectomy	67 (8.61)	69 (9.02)
Uterine-incision delivery	62 (7.97)	65 (8.50)
Liver and gall surgery		
Laparoscopic cholecystectomy	71 (9.13)	68 (8.89)

*χ* ^2^ test, *P* = 0.923, *vs* paper-based guideline.

### Time of the initial dose and the duration of antimicrobial agent

To prevent surgical site infection, antimicrobial agents were mostly administrated within 30 min and 2 h before incision. There were 656 patients (85.75%) intervened with *DRUGS* and 256 cases (32.90%) with paper-based guidelines, which showed that the time of the initial dose of antimicrobial agent changed significantly by *χ* ^2^ test (*P<0.05*). Furthermore, the dose used in *DRUGS* group (92.94%) was significantly higher than the paper-based guidelines group (87.40%) in the pre-operation. There was also significantly different in the time of the initial dose of antimicrobial agent among the three stages of operation (*P<0.05*).

The time of antibiotic prophylaxis was shown in Table 3. The duration of administration in each surgical department was decreased significantly between pre-and post-intervention (*P*<0.05) by *t*-test (or *t*'-test)(Table 4). The number of patients who withdrawn antimicrobial agents within 24 h in the clean wounds incision was 547 (91.62%) and 91 (14.97%) for without and with *DRUGS*, respectively (P<0.05).

**Table 3:** Time of the initial dose of antimicrobial agent among pre-and post-intervention.

Time	Paper-based guidelines n(%)	DRUGS n(%)
Pre-operation		
t≤30 min	49 (6.30)	12 (1.57)*
30 min<t≤2 h	256 (32.90)	656 (85.75)**
2 h<t≤24 h	360 (46.27)	37 (4.84)**
t≥2 d	15 (1.93)	6 (0.78)*
Middle- operation	61 (7.84)	34 (4.44)*
Post-operation	37 (4.76)	20 (2.61)*

*χ* ^2^ test,

* P<0.05,

** P<0.01 vs. paper-based guidelines.

**Table 4:** The duration of administration in each surgical department.

	Paper-based guidelines (day, mean±(d))	DRUGS (day, mean±(d))
General surgery	2.57±1.45	0.46±0.21**
Stomach intestine surgery	3.01±2.16	0.59±0.23**
Orthopedics	3.27±2.31	0.88±0.41**
Gynaecology and obstetrics	4.87±1.86	2.24±1.02*
Liver and gall surgery	2.73±0.84	0.82±0.45*

*t*-test (or *t*'-test),

* P<0.05,

** P<0.01 vs. paper-based guidelines.

### Changes of average cost and hospitalization days of patients

A total of 19 kinds of antibiotics were involved in this study, the leading three places on the list of frequency were piperacillin and sulbactam sodium, cefathiamidine, and cefoperazone, while after post-intervention, the list of frequency changed to cefazolin, cefathiamidine and cefoperazone. Compared to the paper-based guidelines, the intervention was followed by a significant increase in the proportion of cefazolin, 89.71% DRUGS vs. 15.43% (P<0.05). The average hospital stay was significantly decreased, and the average cost of antimicrobial agents after the intervention decreased significantly (P<0.05). The average cost of antibiotics was ¥(3481.36±2584.46) with paper-based guidelines and ¥(1693.39±1478.27) with *DRUGS*, respectively. The changes of average cost and hospitalization days are shown in Table 5. However, the incidences of surgical site infections and adverse drug events or reactions were not significantly different in the post-intervention group (Table 5).

**Table 5:** Changes of average cost and hospitalization days in *DRUGS* (n = 765) and paper-based guidelines (n = 778).

	ACH		ACM		ACA		AHD	
	PBG	*DRUGS*	PBG	*DRUGS*	PBG	*DRUGS*	PBG	*DRUGS*
**Myomectomy**	17576	15142	6806	6104	4125	2308	6	3
**Uterine-incision delivery**	12569	8692	4980	3140	3674	1781	5.7	3.94
**Inguinal hernioplasty**	18464	16481	6866.3	3266.3	725.2	283.8	4	1.2
**Thyroid resection**	9062.47	8813.46	3566	2286	504.1	47.2	5.3	0.8
**Discectomy**	28621.5	21251.3	8474	4055	3253.7	1156.2	13.3	3.7
**Laparoscopic cholecystectomy**	11544.6	10124.4	4520.9	3941	1242.5	962.1	6.3	1
**Breast mass resection**	131561	11326	5213.4	4252	2342.7	1172.4	2	0.58
**Total hip Replacement**	75251	61663	29083	14253	8547.6	5088	15.7	5.2
**Internal fixation of fracture**	35623	22948	10842	4988	6823	2986	3.7	2.75
**Excision of meniscus of knee**	26694.6	21396.5	5625.94	3751	3575.8	1149.2	8	3.24

PBG: paper-based guidelines.ACH: average cost of hospitalization; ACM: average cost of medicine; ACA: average cost of antibiotics; AHD: average hospitalization days.

### Overall physicians' adherence rate to the surgical antibiotic prophylaxis guideline for both control and intervention group

Totally, 1543 surgical operations were recorded in this study, where 778 followed the paper-based guidelines and 765 followed *DRUGS*. The physicians' adherence rate to the surgical antibiotic prophylaxis was significantly increased 11.18%(87/778) with the paper-based guidelines versus 69.93%(535/765) with DRUGS (P<0.01).

## Discussion

SSIs are common risks in surgical procedure. Therefore, prophylactic antibiotics was still used in the post-operative to reduce the risk of SSIs [21], [22] despite lack of evidence of efficacy and rule about discontinuation of antibiotics 24 h after operation in guidelines [23]. In order to prevent from post-operation infection, surgeons used to antibiotics according to papers even to their own experience, which might lead to abuse of antibiotics. It was reported that adherence rate to guidelines was about 44–46% in ambulatory patients with community-acquired pneumonia [24]. In the hospitals of China, overuse of antibiotics after surgical procedure is becoming a noteworthy issue.

In the proceeding of diagnose and treatment, drugs for patients are not prescribed on the basis of treatment guidelines and the individual situation, but on their randomized self-experiences, even on the amount of rebates from pharmaceutical companies, which were the major reasons that led to overly expensive medical service in China. It was very common that the cost of treatment for the same disease in differed several times or in different hospitals. One patient in Beijing saw the doctors in four hospitals with the same medical complaint [31], and obtained four prescriptions to an expert panel, which were masked the names of hospitals and doctors, to make an evaluation. Astonishingly, in the capital city of China, only one prescription was appropriate for the case. While superfluous improper drugs were list on the other three prescription. 10 kinds of diseases are included in our study. The average cost of antibiotics was ¥(3481.36±2584.46) for paper-based guidelines and ¥(1693.39±1478.27) for *DRUGS*, respectively.

To promote clinicians' adherence to the guidelines, we distributed them in booklet form in all the wards of our hospital. However, this way of dissemination does not ensure that information will be read and considered by the clinicians. Grimshaw and Russel [25] claimed that the more overtly educational dissemination strategy, the greater the likelihood that guidelines will be adopted and the more lasting their impact. So we developed *DRUGS* inserted into the Computerized Physician Order Entry System. When the clinicians prescribe on the computer, the pop-up box will remind and assist them using antibiotics rationally.

Inappropriate prophylaxis would slow recovery, prolong hospital stay, increase antibiotic resistance and costs of antimicrobial agents, and so on. In this study, we investigated whether the *DRUGS* system improved the rate of the surgeons' adherence to the antibiotic guidelines during prevention from SSI. In the period using paper-based guidelines, only 15.43% of patients were prescribed antibiotic categories and 32.9% of patients were administrated according to time course recommended by the guidelines during antibiotic prophylaxis of surgical operations. It was reported that the most predictable impact was achieved when the guidelines are made available through computer-based reminders that are integrated into the clinician's workflow and provide therapeutic information to prescribers ‘just-in-time’ to influence the decision at the time of clinical decision [13], [14], [15], [26]. Shojania [27] developed a system in which the CDC recommendations for vancomycin use were presented online to the surgeon at the time of drug ordering on a hospital's computer-based order entry system, which found that surgeons who were randomly assigned to receive the intervention wrote 32% fewer orders for vancomycin compared with control group. In addition, the duration of vancomycin therapy in the intervention group was 36% lower than that of control surgeon. In our study, the intervention with *DRUGS* also showed exciting results 89.71% and 85.75% of the patients were reasonably administrated antibiotic prophylaxis of surgical operations in the choice of antibiotic categories and the time of administration, which was much higher than those of the group with paper-based guidelines.

In addition to the global improvement of surgeons' adherence to guidelines associated with the *DRUGS*, the guidelines effect on the gynaecology and obstetrics department appeared to be relatively sustained in the later period. It still keeps longer duration of administration than other department in the antibiotic orders. This might be due to the low adherence rate of physicians of gynaecology and obstetrics than other.

The increased availability of antibiotic guidelines at the time of prescription, combined with a periodical reinforcement educational round, was associated with an enhanced adherence of surgeons to the guidelines. Therefore, compulsory training courses on the infection declaration process and the computerized antibiotic guidelines have been performing every three month by the pharmacy department and infection control unit to the newly recruited doctors in our hospital, which will assist them to use DRUGS system fully and prescribing reasonably.

Another recommended method to improve the quality of clinical pharmacists to antibiotic prescribing [28], [29]. As part of a multidisciplinary team, their feedback will help to enhance the appropriateness of drug prescribing. Ansari [30] reported that the effectiveness on the quality of antibiotic prescribing of an immediate concurrent, patient-specific feedback of information to the prescribers who were contacted by the clinical pharmacist while their patient was still being treated. However, that concurrent prescription review and feedback by clinical pharmacists is very high cost and hard to apply in Chinese hospitals with limited human resources in the pharmacy department. For example, there are only 10 clinical pharmacists in our hospital, which is greatly insufficient to provide concurrent prescription review and feedback for two hundred surgeons. So we prefer to *DRUGS* system for improving descriptions of antibiotics reasonably.

There were several limitations in our study. Firstly, the *DRUGS* system only involves several specific diseases/operations.In order to minimize the potential bias of current research,we will investigation and validation with more diseases/operations in further study. Secondly, because of the lack of enough experience and education for the pharmacists, it may have prevented the detection of more interventions. Therefore, a longer training period for the researcher might have produced better results. Thirdly, the patients in this study were mainly came from urban area in Xi'an city (the most economically developed city in the northwesten China).So, there may be a lack of statistical power to represent more significant differences for patients in other areas. Thus, to generalize the results, larger sample sizes and wider areas would be needed. Finally, the limited number of cases in some kinds of operations may also impact the reliability of the conclusion of this study, such as thyroid resection only has 100 participants, 53 for PBG and 47 for *DRUGS*.

In conclusion, we developed a system embedding clinical practice guidelines in a computerized surgeon order entry system, *DRUGS*, which was applicable to hospitals in China providing with such an information technology. As shown by the results obtained in this study, it remains, however, that additional reinforcing interventions, such as immediate concurrent feedback by hospital pharmacists, appear to be necessary for further enhancing appropriateness of antibiotic prescribing.
